# A218 ASSOCIATIONS BETWEEN ADHERENCE TO LITERATURE-DERIVED DIETARY INDICES AND PRE-DISEASE BIOMARKERS: IMPLICATIONS FOR CROHN’S DISEASE PREVENTION

**DOI:** 10.1093/jcag/gwac036.218

**Published:** 2023-03-07

**Authors:** A Neustaeter, S -H Lee, M Xue, H Leibovitzh, K Madsen, J B Meddings, O Espin-Garcia, A M Griffiths, P Moayyedi, A H Steinhart, R Panancionne, H Huynh, K Jacobson, G Aumais, D Mack, C Bernstein, J K Marshall, W Xu, W Turpin, K Croitoru

**Affiliations:** 1 University of Toronto, Toronto; 2 University of Alberta, Edmonton; 3 Cumming School of Medicine, Calgary; 4 The Hospital for Sick Children,, Toronto; 5 McMaster University, Hamilton; 6 University of Calgary; 7 University of Alberta, Calgary; 8 University of British Columbia, Vancouver; 9 Montreal University, Montreal; 10 University of Ottawa, Ottawa; 11 University of Manitoba, Winnipeg, Canada

## Abstract

**Background:**

The incidence of Crohn’s disease (CD) is increasing globally, indicating a significant environmental influence such as diet. A plethora of dietary adherence (DA) patterns exist in the literature: the Mediterranean Diet (MD), Empirical Dietary Inflammatory Pattern (EDIP), Specific Carbohydrate Diet (SCD), and low Fermentable Oligosaccharide, Disaccharide, Monosaccharide, or Polyol diet (FODMAP) are all potential candidates to maintain a reduced level of inflammation, improving gastrointestinal function. Contrary, the Westernized diet (WD) is generally reported as a diet promoting inflammation in humans.

**Purpose:**

To determine if DA to literature-derived dietary indices in a cohort of first-degree relatives (FDRs) of CD patients can modulate pre-disease biomarkers.

**Method:**

We used food frequency questionnaire (FFQ) data from 2,696 healthy FDR subjects of the Crohn’s Colitis Canada- Genes, Environment, Microbial (CCC-GEM) project. We rederived each of the following scores using our FFQ data, utilizing originally described methods for the MD, EDIP, SCD, low FODMAP, and WD to obtain DA. Each diet was correlated pairwise via Kendall’s Tau.

We fit multivariable regression models to identify the association of DA (top quintile vs remaining) and: i) intestinal permeability using urinary fractional excretion of lactulose to mannitol ratio (LMR), LMR≥0.03 defined abnormal; ii) subclinical inflammation using fecal calprotectin (FCP) measured with BÜHLMANN fCAL® ELISA, FCP≥250µg/g defined inflammation; and iii) fecal microbiome richness and composition using 16S rRNA sequencing. Two-sided p<0.05 for primary and q<0.05 for secondary analysis defined significance.

**Result(s):**

There were positive correlations between the MD, SCD, and low FODMAP, these diets negatively correlated with the WD. The EDIP negatively correlated with the SCD and low FODMAP, did not correlate with the MD, and positively correlated with the WD. No diet was associated with abnormal LMR or FCP. Only the SCD was associated with increased microbial richness (q=0.03). All diets were associated with microbial genera: the MD (n=18 taxa, (2.0^-7^<q-values< 0.04), EDIP (n=9, [2.8^-4^-0.05]), SCD (n=13, [3.7^-11^-0.05]), low FODMAP (n=14, [1.3^-7^-0.05]), and WD (n=1, [0.03]).

**Conclusion(s):**

This study shows that literature-derived dietary indices correlate generally with each other, yet none were not associated with abnormal LMR or FCP. However, we found that diet can impact microbiome richness and composition. Thus, it is tempting to speculate that diet is a possible intervention capable of maintain microbiome homeostasis to reduce future risk of CD.

Submitted on behalf of the CCC-GEM consortium.

**Funding**

Crohn’s and Colitis Canada Genetics Environment Microbial (CCC-GEM) III

The Leona M. and Harry B. Helmsley Charitable Trust

Kenneth Croitoru is the recipient of the Canada Research Chair in Inflammatory Bowel Disease

**Disclosure of Interest:**

None Declared

